# Healthcare Utilization Disparities Among Older Adults With and Without Cognitive Impairment: Health and Retirement Study Findings

**DOI:** 10.1007/s10823-025-09553-w

**Published:** 2026-02-09

**Authors:** Zahra Rahemi, Sophia Z. Shalhout, Juanita-Dawne R. Bacsu, Darina V. Petrovsky, Preeti Pushpalata Zanwar, Swann Arp Adams

**Affiliations:** 1 School of Nursing, Clemson University, Clemson, SC 29634-0743 USA; 2 Division of Surgical Oncology, Department of Otolaryngology- Head and Neck Surgery, Mike Toth Cancer Center, Mass Eye and Ear, Boston, MA USA; 3 Department of Otolaryngology- Head and Neck Surgery, Harvard Medical School, Boston, MA USA; 4 School of Nursing, Thompson Rivers University, 805 TRU Way, Kamloops, BC V2C 0C8 Canada; 5 Division of Women, Children and Families, Duke University School of Nursing, 307 Trent Drive Box 3322, Durham, NC 27710 USA; 6 Center for Healthy Aging Behaviors and Longitudinal Investigations, University of Chicago, Chicago, IL, 60637, USA, Chicago, IL USA; 7 College of Nursing and the, Department of Epidemiology & Biostatistics, University of South Carolina, Columbia, SC 29208 USA

**Keywords:** Health and Retirement Study, Race, Ethnicity, Healthcare use, Hospital, Nursing home, Cognition

## Abstract

The purpose of this study was to determine the healthcare utilization patterns in a national sample of older adults across several factors (ethnicity, gender, race, education) with normal and dementia/impaired cognition. We used datasets from the Health and Retirement Study (HRS, 2018) to evaluate healthcare utilization, including metrics such as hospital and nursing home stays, hospice care, and the number of visits to the doctor. Logistic models were used to predict healthcare utilization separately in those with normal cognition and dementia. Our final sample comprised 15,607 adults (mean age: 65.2 normal cognition, mean age 71.5 dementia). Hispanics with normal cognition were less likely to stay in a hospital than non-Hispanic respondents (OR: 0.52–0.71, p < 0.01). Being female was associated with a higher risk for shorter nursing home days (OR: 1.41, p < 0.01) and doctor visits (OR: 1.63–2, p < 0.01) in cognitively normal older adults. Being female was associated with a lower risk for hospital stay in those with dementia (OR: 0.50–0.78, p < 0.01). Respondents identifying as Black or other races with dementia were less likely to experience nursing home days (OR: 0.42, p < 0.04). Black respondents with normal cognition were less likely to experience doctor visits (OR: 0.32–0.37, p < 0.01). Those with more than a high school education in both groups were more likely to experience doctors’ visits. The study points to the continued disparities in healthcare utilization linked to participants’ characteristics and cognition.

Cognitive impairment due to dementia has become a pressing public health concern. An estimated 6.7 million Americans aged 65 and older live with Alzheimer’s disease and related dementia (ADRD) (Alzheimer’s Association, 2024). This number is projected to more than double by 2060 (Rajan et al., 2021), disproportionately affecting minority groups, including Black, Hispanic, and underserved older adults (Garcia et al., 2019; Rahemi et al., 2022; Wiese et al., 2023). For example, Hispanic individuals are about 1.5 times more likely to develop ADRD than non-Hispanic Whites (Alzheimer’s Association, 2024). Research indicates that Black individuals aged 50 years and older are two to three times more likely than Caucasians to develop dementia (Garcia et al., 2019). In addition, racial and ethnic minorities typically experience suboptimal quality in care, limited access to healthcare (Mcmaughan et al., 2020), and structural inequities (Peterson et al., 2024) while requiring more frequent and complex healthcare services. This may potentially result in higher rates of healthcare utilization and increased medical costs (Charron-Chénier & Mueller, 2018; Pereira et al., 2022). These findings highlight the crucial need for research to tackle the growing challenges in healthcare utilization and disparities among diverse older adults with cognitive impairment (Pereira et al., 2022; Tappen et al., 2017).

An emerging body of literature shows persistent racial and ethnic disparities in healthcare utilization among older adults with neurodegenerative diseases (Borson et al., 2022; Hinton et al., 2024; Lusk et al., 2023; Palms et al., 2025; Rahemi & Parker, 2021; Rahemi & Williams, 2020; Uddin et al., 2025). A recent scoping review found that minority racial and ethnic groups are less likely to get a timely dementia diagnosis, receive dementia medications and treatment options, use hospice care, and have an increased risk of hospitalization (Hinton et al., 2024). Likewise, Lusk and colleagues reported that Black beneficiaries were significantly less likely than White beneficiaries to receive hospice care, physical therapy, and dementia-related medications, even after adjusting for key covariates (Lusk et al., 2023). Moreover, Palms and colleagues note that racial and ethnic minority groups face disproportionate structural disparities that impact dementia care related to factors such as access to education, financial stability, social support, built environment, cultural beliefs, and access to quality health care (Palms et al., 2025).

Beyond conceptions of culture rooted in race and ethnicity, a growing body of research addresses the influence of cultural context related to geography in understanding how people utilize healthcare services (Bacsu et al., 2019; Cohen & Greaney, 2022; Lin et al., 2022; Rahemi & Parker, 2021). For example, cultural context and geography influence healthcare utilization by impacting healthcare behaviors, beliefs, decision-making, and access to healthcare and support services (Lin et al., 2022). Specifically, Bacsu and colleagues note that rural communities often experience unique disparities in accessing cognitive healthcare compounded by limited education, transportation, healthcare specialists, finances, as well as challenges related to dementia-related stigma and misinformation (Bacsu et al., 2019). Greenwood-Ericksen and Kocher note that rural areas have a higher rate of unnecessary emergency department visits, partly due to the dispersed geography and a lack of rural healthcare professionals. Other factors like waiting too long to seek care, cultural beliefs, feeling ashamed, gender (Greenwood-Ericksen & Kocher, 2019; Wiese et al., 2023), health literacy, insurance coverage, and resource availability can also make it harder for people to receive the right care (Chen et al., 2016; Rahemi, 2019; Rahemi & Williams, 2020). As a result, minority groups and underserved individuals often experience poor health outcomes, exacerbating systemic healthcare disparities and inequities in health outcomes (Charron-Chénier & Mueller, 2018; Wiese et al., 2023).

Healthcare costs are notably higher for racial minority individuals with ADRD compared to minority individuals with normal cognition and White individuals with and without ADRD (Cooper et al., 2010; Pereira et al., 2022). Additionally, older adults with ADRD exhibit higher rates of emergency department visits, hospitalizations, and prolonged hospital stays than their counterparts without ADRD (Benner et al., 2018; Hunt et al., 2018; Kent et al., 2019). The increased rates of healthcare utilization, in turn, are associated with increased mortality rates, higher risks of fall-related injuries, earlier admissions to nursing homes, and changes in both cognitive and physical health (Colligan et al., 2017; Pereira et al., 2022). To address these disparities, some scholars emphasize the role of advance care planning (ACP), which involves proactively communicating healthcare decisions before a person loses decision-making capacity (McMahan et al., 2021).

Utilizing the past 2014 HRS dataset, we previously demonstrated that participants who were younger, Hispanic, Black, had lower levels of education, or resided in rural areas were less likely to complete ACP (Rahemi et al., 2024). Furthermore, those individuals with ACP were more likely to experience longer stays in hospitals, nursing homes, and home healthcare (Rahemi et al., 2023a). Ongoing research is essential to tackle critical factors, including race, ethnicity, sex, and gender, and the role of ACP, as well as their intersections. These factors contribute to the increasing healthcare challenges and disparities observed in diverse older adults, particularly those with cognitive impairment. Research in this field concerning ADRD and cognitive disorders is limited (Hinton et al., 2024; Pereira et al., 2022). A recent review of 96 randomized controlled trials to support cognition related to dementia (n = 37,278) identified that only 39 trials (39.4%) included ethnicity, and only 11.4% (95% CI, 7.5 to 15.9%) of all participants were non‐Caucasian (Vyas et al., 2018). However, as the American population becomes more racially, ethnically, and culturally diverse, there is a critical need for research to examine disparities in healthcare utilization for diverse people living with ADRD and cognitive disorders (Hinton et al., 2024). Although extensive literature on healthcare utilization exists, there remains a paucity of information related to service utilization among diverse racial, ethnic, and cultural groups, especially in terms of exploring varying levels of cognitive function.

To gain a more comprehensive understanding of the disparities in care for ADRD across racial/ethnic, cultural, and cognitive groups, we analyzed predictors of healthcare utilization in a national sample of older adults. Our focus was on key factors, including age, gender, race, ethnicity, education, marital status, and rural location. We further sought to determine whether any significant predictors found with the analysis were mediated by the ACP measures of having a living will, durable power of attorney for healthcare, or both. We utilized Health and Retirement Study (HRS, 2018) datasets to evaluate healthcare utilization among a diverse population of U.S. older adults. This involved analyzing data on the total length of hospital stays, nursing home stays, hospice care, and the frequency of doctor visits over the past two years.

This study addresses the need for research on ongoing challenges related to healthcare disparities and utilization among diverse older adults with cognitive impairment, as highlighted by recent evidence showing persistent disparities and rising related medical costs (Charron-Chénier & Mueller, 2018; Pereira et al., 2022; Rahemi & Jarrín, 2023). A growing body of research has focused on healthcare utilization among diverse populations (Borson et al., 2022; Lusk et al., 2023; Pereira et al., 2022; Rahemi et al., 2023a, 2023b). However, our study is both innovative and comprehensive in examining a range of healthcare utilization patterns across diverse racial, ethnic, and cultural groups, while also accounting for the role of ACP and varying levels of cognitive function. The conceptual framework guiding this study is based on the Andersen Behavioral Model of Health Service Use, which identifies three categories of factors that impact individuals’ healthcare utilization: individual characteristics, contextual characteristics, and health behaviors (Andersen, 1995). Our investigation was focused on assessing the predictive relationship between the contextual characteristics (e.g. neighborhood cohesion, living alone, discrimination), individual characteristics (e.g. race, ethnicity, gender, insurance, health status), and health behaviors (utilization of hospital, hospice, nursing home, and doctor office services) (Fig. 1).

**Fig. 1:**
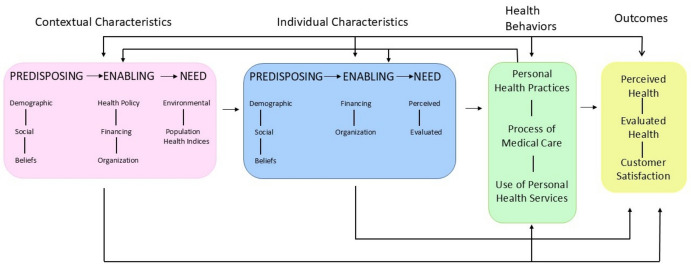
Conceptual framework based on the andersen behavioral model of health service use

## Materials and Methods

This observational, cross-sectional study is a secondary analysis of data from the Health and Retirement Survey (HRS), a nationally representative survey of respondents aged 51 years and older (Sonnega et al., 2014). The HRS utilizes a probability sampling method to ensure a representative demographic sample, with an oversampling of African American, Hispanic, and Floridian participants. For our study, we analyzed a sample of 15,607 respondents from the HRS 2018 survey (wave 14). Our analysis incorporated data from the harmonized HRS version B pertaining to end-of-life topics and the 2018 Rand HRS Longitudinal version 2. As our research utilized secondary data analysis of de-identified data, it was exempt from review by the university’s Institutional Review Board.

### Measures

For analysis, the HRS respondents were categorized into two groups using the Langa-Weir approach: dementia/impaired cognition (scores of 1–11) and normal cognition (scores of 12 or higher) (Langa et al., 2017). Previous work with the HRS dataset has demonstrated significant interaction with the cognition group (Rahemi et al., 2024). In examining the distributions of our four outcome healthcare utilization variables (hospital days, hospice days, nursing days, and doctor visits), we realized the distributions were highly skewed with many ‘0 days’ observations. We attempted to fit several different models, including zero-inflated Poisson, zero-inflated negative binomial, and various dependent variable transformations, using the chi-square goodness-of-fit test. No single model was a good fit for all four outcomes. Consequently, we ultimately grouped these variables into three or four categories: never, low, moderate, and high utilization (hospital stay and doctor visits) or never, some, and high utilization (nursing home and hospice care) to enhance readability and interpretation of our findings.

Our literature review could find no commonly used cut points; therefore, we based our cut points on natural breaks in the frequency distribution of each variable using a histogram plot for each outcome (Nuzzo, 2019). We aimed to align these points across variables to the greatest extent possible. Table 1 details the division and cut points for each healthcare variable. Using a data-driven approach, we inspected histograms and box plots to identify these breaks and employed gap statistics to determine the optimal number of bins or clusters, ensuring significant gaps between clusters.

**Table 1:** Healthcare utilization categories and their cut points

Utilization Type	Never	Low (# of days)	Medium (# of days)	High (# of days)
Hospital Days	0	1–7	8–15	16 +
Doctor Visits	0	1–6 (visits)	8–25 (visits)	26 + (visits)
	Never	Some	High	
Nursing Home	0	1–119	120 +	
Hospice Care	0	1–15	16 +	

For the duration of hospitalization within the preceding two years, the groups were never (0), low (1–7 days), moderate (8–15 days), and high (16 + days). For nursing home care in the previous two years, the cut points were never (0), low (1–30 days), moderate (31–199 days), and high (120 + days). The number of doctor visits in the previous two years collapsed into never (0), low (1–6 visits), moderate (8–25 visits), and high (26 + visits).

Race was assessed by a single question, “What race do you consider yourself to be: White, Black or African American, American Indian, Alaska Native, Asian, Native Hawaiian, Pacific Islander, or something else? and at the time of entry into the HRS cohort study. Gender was assessed by the survey administrator “Mark sex of respondent” at the time of entry into the HRS cohort study. Rurality was based upon the patient’s residence, rural or urban. For the ACP mediation analysis, living will and durable power of attorney for healthcare were captured as present (yes/no). For the combination variable, respondents were classified as no ACP measure (neither living will or durable power of attorney for healthcare), at least one ACP measure (either living will or durable power of attorney for healthcare), or having both ACP measures.

### Statistical Analysis

All analyses were performed using SAS v9.4. An alpha level of less than or equal to 0.05 was set for statistical significance. Given the potential for bias from the differing healthcare needs of individuals with dementia or impaired cognition compared to those with normal cognition. All analyses were stratified by cognition group as described previously. Due to the complex sampling design of the HRS, participant sample weights were applied to all descriptive statistics to ensure nationally representative estimates. Weighted frequencies and means were calculated for the appropriate variables. Weighted sample t-tests and Rao-Scott chi-square tests were employed to determine significant differences between cognition groups (dementia/impaired cognition versus normal cognition).

Previous analyses have shown that using sample weights in multivariable modeling can introduce bias into the estimates (Winship & Radbill, 1994); therefore, we did not incorporate these weights in any of the model results presented. The Proc logistic function was used with the glogit option to conduct polytomous logistic regression models. Each healthcare utilization variable was included as a dependent variable and demographic variables were incorporated as independent variables. The ‘never’ utilization was the reference group for each model. All polytomous logistic models were stratified by cognition group (dementia/impaired cognition versus normal cognition) to facilitate the comparison of various predictors.

For the mediation analysis, we employed the Baron and Kenny method (Baron & Kenny, 1986). Initially, we assessed whether each ACP variable was associated with the SDOH variable and the healthcare utilization outcome. If both conditions were met, a single model was constructed, including the advance care planning variable, the relevant SDOH variable, and the healthcare outcome variable as the dependent variable. Mediation was considered to exist if the previously significant SDOH variable no longer achieved statistical significance in this combined model.

## Results

Table 2 describes and compares the demographic characteristics of the dementia/impaired cognition group in relation to the normal cognition group. In contrast to the normal cognition group, the dementia/impaired cognition group participants had significantly less education, were more likely to be from a racial/ethnic minority community, single, and live in a rural geographical area. Compared to the normal cognition group, those in the dementia/impaired cognition group utilized a greater proportion of hospital and nursing home care and were more likely to have fewer low and medium doctor visits and increased high and no doctor visits. In the dementia/impaired cognition group, the mean age was significantly greater than the normal cognition group (71.5 compared to 65.2).

**Table 2:** Descriptive statistics for the 2018 heath retirement survey cohort* By Cognition Group (HRS, 2018)

	Normal Cognition N = 12,540 Weighted % (n)	Dementia/Impaired Cognition N = 3067 Weighted % (n)	P-Value
Education			
< 12 years	36.1 (5215)	69.4 (2254)	< 0.01
13 + years	63.9 (7374)	30.6 (825)	
Sex			
Male	45.9 (6503)	45.9 (1297)	0.93
Female	54.1 (9165)	54.1 (1782)	
Race			
White	82.3 (8784)	63.6 (1591)	< 0.01
Black/Other	17.7 (3756)	36.4 (1476)	
Ethnicity			
Hispanic	9.0 (1811)	18.2 (699)	< 0.01
Non-Hispanic	91.0 (10,759)	81.8 (2371)	
Marital Status			
Married/Living with a Partner	67.0 (7829)	49.0 (1496_	< 0.01
Single/Widowed	33.0(4760)	51.0 (1583)	
Rurality			
Rural	31.4 (3672)	36.2 (1034)	< 0.01
Urban	68.6 (8917)	63.8 (2045)	
Hospitalization Days Categories			
None (0 days)	79.9 (9762)	71.4 (2151)	< 0.01
Low (1–7 days)	15.7 (2081)	20.0 (579)	
Medium (7–15 days)	2.6 (383)	4.7 (143)	
High (16 + days)	1.8 (252)	3.9 (110)	
Hospice Care Days Categories†			
None (0 days)	79.2 (3724)	80.5 (4217)	0.05
Some (1–15 days)	13.4 (629)	13.3 (694)	
High (16 + days)	7.4 (347)	6.2 (323)	
Nursing Home Days Categories			
None (0 days)	97.9 (12,237)	94.7 (2858)	< 0.01
Some (1–119 days)	1.7 (222)	3.2 (103)	
High (120 + days)	0.4 (49)	2.1 (53)	
Doctors Visits Categories			
None (0 visits)	8.9 (1168)	16.9 (522)	< 0.01
Low (1–6 visits)	51.9 (6022)	48.3 (1252)	
Medium (7–25 visits)	33.7 (4076)	28.9 (742)	
High (26 + visits)	5.5 (644)	5.9 (143)	
	**Mean (SE)**	**Mean (SE)**	
Age (yrs)	65.2 (0.2)	71.5 (0.4)	< 0.01

*Sampling weights are not provided for deceased participants so only alive respondents are included for weighted analyses in this table

†This variable is derived from the exit interview survey of deceased participants, which does not have sample weights. Thus, variance estimates have not been adjusted for the sampling design

### Hospital Stay

In the normal cognition group, when examining each level of hospitalization to those who had never been hospitalized, Hispanics were significantly less likely to have low, moderate, and high hospital stays compared to non-Hispanic participants. Older age was significantly associated with hospitalization at each level of utilization.

Among those with dementia/impaired cognition, when examining each level of hospital use, Hispanics were significantly less likely to utilize low and moderate lengths of stay compared to the non-Hispanic group. Females were significantly less likely to have hospital stays. Older age was associated with a significant trend going from short to long stays; however, only the point estimate for the short hospital stays reached statistical significance, with older age demonstrating a greater likelihood of a short hospital stay (Table 3).

**Table 3:** Hospital days prediction model*, health retirement survey, 2018

		Normal Cognition				Dementia/Impaired Cognition			
		Low vs. Never	Moderate vs. Never	High vs. Never	p-value for trend	Low vs. Never	Moderate vs. Never	High vs. Never	p-value for trend
Race	White	1.00	1.00	1.00	0.67	1.00	1.00	1.00	0.36
	Black/Other	NS	NS	NS		NS	NS	NS	
Ethnicity	Not Hispanic	1.00	1.00	1.00	** < 0.01**	1.00	1.00	1.00	** < 0.01**
	Hispanic	**0.71 (0.61, 0.83)**	**0.66 (0.47, 0.94)**	**0.52 (0.33, 0.83)**		**0.74 (0.58, 0.95)**	**0.61 (0.37, 0.98)**	0.58 (0.34, 1.00)	
Rural	No	1.00	1.00	1.00	0.21	1.00	1.00	1.00	0.84
	Yes	NS	NS	NS		NS	NS	NS	
Marital Status	Single	1.00	1.00	1.00	0.14	1.00	1.00	1.00	0.76
	Married	NS	NS	NS		NS	NS	NS	
Sex	Male	1.00	1.00	1.00	0.19	1.00	1.00	1.00	** < 0.01**
	Female	NS	NS	NS		**0.78 (0.64, 0.95)**	0.88 (0.61, 1.27)	**0.50 (0.33, 0.75)**	
Education	< HS	1.00	1.00	1.00	0.95	1.00	1.00	1.00	0.57
	> HS	NS	NS	NS		NS	NS	NS	
Age		**1.03 (1.02, 1.03)**	**1.03 (1.02, 1.04)**	**1.02 (1.00, 1.03)**	** < 0.01**	**1.02 (1.01, 1.03)**	1.01 (0.99, 1.03)	1.00 (0.98, 1.02)	** < 0.01**

Odds ratios (95% CI)

^*^ Model adjusted for number of chronic conditions and physical impairment

Bold = Statistical significance

NS = Not significant. NS was indicated when all results across utilization levels were not significant

The length of hospitalization in the previous two years: never (0), low (1–7 days), moderate (8–15 days), and high (16 + days)

### Nursing Home Stay

In the normal cognition group, when examining each level of stay, married individuals were significantly less likely to utilize all lengths of nursing home stays, while older participants were more likely to utilize all levels of nursing home stays. Women were significantly more likely to experience shorter stays in the nursing home while significantly less likely to utilize longer stays (see Table 3- trend test). Hispanic participants were significantly less likely to utilize some nursing home care compared to non-Hispanic individuals (Table 4).

**Table 4:** Nursing home days prediction model*, health retirement survey, 2018

		Normal Cognition			Dementia/Impaired Cognition		
		Some vs. Never	High vs. Never	p-value for trend	Some vs. Never	High vs. Never	p-value for trend
Race	White	1.00	1.00	0.06	1.00	1.00	** < 0.04**
	Black/Other	NS	NS		0.75 (0.48, 1.18)	**0.42 (0.21, 0.88)**	
Ethnicity	Not Hispanic	1.00	1.00	**0.03**	1.00	1.00	** < 0.01**
	Hispanic	**0.46 (0.26, 0.83)**	1.00 (0.34, 2.96)		**0.23 (0.10, 0.52)**	**0.25 (0.07, 0.82)**	
Rural	No	1.00	1.00	0.52	1.00	1.00	0.20
	Yes	NS	NS		NS	NS	
Marital Status	Single	1.00	1.00	** < 0.01**	1.00	1.00	** < 0.01**
	Married	**0.64 (0.47, 0.85)**	**0.23 (0.11, 0.48)**		0.85 (0.55, 1.31)	**0.26 (0.12, 0.57)**	
Sex	Male	1.00	1.00	** < 0.01**	1.00	1.00	0.20
	Female	**1.41 (1.03, 1.92)**	**0.49 (0.26, 0.91)**		NS	NS	
Education	< HS	1.00	1.00	0.11	1.00	1.00	0.81
	> HS	NS	NS		NS	NS	
Age		**1.05 (1.03, 1.06)**	**1.10 (1.07, 1.13)**	** < 0.01**	**1.04 (1.02, 1.06)**	**1.07 (1.04, 1.10)**	** < 0.01**

Odds ratios (95% CI)

^*^ Model adjusted for the number of chronic conditions and physical impairment

Bold = Statistical significance

NS = Not significant. NS was indicated when all results across utilization levels were not significant

Nursing home care in the previous two years: never (0), some (1–199 days), and high (120 + days)

Among dementia/impaired cognition and normal cognition groups, marital status (p < 0.01) and age had a similar trend effect on nursing home utilization: lower utilization for married and higher utilization for older individuals. In contrast to the normal cognition group, in the dementia/impaired cognition group, race and ethnicity also proved to have a significant trend in utilization, while rurality and sex did not have a significant trend. Based upon the point estimate testing, those of Black/other race and Hispanic ethnicity in dementia/impaired cognition group were significantly less likely to utilize high lengths of nursing home stays. Hispanic participants were significantly less likely to utilize some nursing home care compared to non-Hispanic individuals.

### Hospice Care

Among the normal cognition group, only increased age was associated with a significantly increased likelihood of utilizing hospice care (Table 5). Among those with dementia/impaired cognition, individuals who had more education were significantly more likely to have moderate utilization compared to less educated individuals. Furthermore, with increasing age, individuals with impaired cognition or dementia were more likely to utilize a longer length of hospice care (see Table 4- trend test).

**Table 5:** Hospice care prediction model*, health retirement survey, 2018

		Normal Cognition			Dementia/Impaired Cognition			
		Some vs. Never	High vs. Never	p-value for trend		Some vs. Never	High vs. Never	p-value for trend
Race	White	1.00	1.00	0.24		1.00	1.00	0.43
	Black/Other	NS	NS					
Ethnicity	Not Hispanic	1.00	1.00	0.50		1.00	1.00	0.33
	Hispanic	NS	NS			NS	NS	
Marital Status	Single	1.00	1.00	0.19		1.00	1.00	0.66
	Married	NS	NS			NS	NS	
Sex	Male	1.00	1.00	0.42		1.00	1.00	0.43
	Female	NS	NS			NS	NS	
Education	< HS	1.00	1.00	0.18		1.00	1.00	** < 0.01**
	> HS	NS	NS			**1.44 (1.18, 1.76)**	0.87 (0.64, 1.20)	
Age		**1.02 (1.01, 1.03)**	1.00 (0.99, 1.02)	** < 0.01**		1.01 (0.99 1.02)	**1.02 (1.01, 1.04)**	**0.01**

Odds ratios (95% CI)

* Model adjusted for physical impairment

Bold = Statistical significance

NS = Not significant. NS was indicated when all results across utilization levels were not significant

The length of hospice care in the previous two years: Never (0), some (1–15), high (16 +)

### Doctor Visits

Among the normal cognition group, race, ethnicity, marital status, sex, education, and age had a significant trend across utilization levels of doctor visits (Table 6- trend test), with respondents from Black/Other race and Hispanic ethnicity less likely to experience doctors’ visits and those who are married, female, older and with greater levels of education were more likely to have doctors’ visits Among dementia/impaired cognition group, only ethnicity and education had significant trends in the utilization of doctor visits. higher education was associated with more doctor visits at all levels. Ethnicity also had a similar effect in both groups, with Hispanic participants less likely to visit the doctor at all frequencies assessed. (Table 6- trend test).

**Table 6:** Doctor’s visits prediction model*, health retirement survey, 2018

		Normal Cognition				Dementia/Impaired Cognition			
		Low vs. Never	Moderate vs. Never	High vs. Never	p-value for trend	Low vs. Never	Moderate vs. Never	High vs. Never	p-value for trend
Race	White	1.00	1.00	1.00	** < 0.01**	1.00	1.00	1.00	0.09
	Black/Other	**0.76 (0.67 0.87)**	**0.67 (0.58, 0.78)**	**0.56 (0.44, 0.70)**		NS	NS	NS	
Ethnicity	Not Hispanic	1.00	1.00	1.00	** < 0.01**	1.00	1.00	1.00	** < 0.01**
	Hispanic	**0.35 (0.30, 0.40)**	**0.28 (0.23, 0.33)**	**0.20 (0.14, 0.27)**		**0.37 (0.29, 0.47)**	**0.32 (0.24, 0.42)**	**0.33 (0.20, 0.53)**	
Rural	No	1.00	1.00	1.00	0.08	1.00	1.00	1.00	0.85
	Yes	NS	NS	NS		NS	NS	NS	
Marital Status	Single	1.00	1.00	1.00	** < 0.01**	1.00	1.00	1.00	0.49
	Married	**1.30 (1.14, 1.49)**	**1.26 (1.09, 1.46)**	**1.39 (1.12, 1.72)**		NS	NS	NS	
Sex	Male	1.00	1.00	1.00	** < 0.01**	1.00	1.00	1.00	0.11
	Female	**1.63 (1.44, 1.85)**	**1.93 (1.68, 2.21)**	**2.00 (1.63, 2.45)**		NS	NS	NS	
Education	< HS	1.00	1.00	1.00	** < 0.01**	1.00	1.00	1.00	** < 0.01**
	> HS	**1.75 (1.54, 1.99)**	**2.12 (1.85, 2.43)**	**1.82 (1.48, 2.22)**		**2.38 (1.80, 3.17)**	**2.90 (2.13, 3.93)**	**3.87 (2.50, 5.99)**	
Age		**1.02 (1.01, 1.03)**	**1.04 (1.03, 1.05)**	**1.02 (1.01, 1.03)**	** < 0.01**	NS	NS	NS	0.17

Odds ratios (95% CI)

^*^ Model adjusted for number of chronic conditions and physical impairment

Bold = Statistical significance

NS = Not significant. NS was indicated when all results across utilization levels were not significant

The number of doctor visits in the previous two years: never (0), low (1–6 visits), moderate (8–25 visits), and high (26 + visits)

### Mediation Analyses

We found no evidence for mediation analysis by our ACP measures for the associations identified between the study variables and healthcare utilization previously noted. Consequently, our models remained unchanged as represented in Tables 3, 4, 5 and 6.

## Discussion

We evaluated healthcare utilization disparities among a nationally representative and diverse cohort of older adults with dementia and normal cognition in the context of age, gender, race, ethnicity, education, marital status, rural location, and ACP. We found that compared to the normal cognition group, participants in the dementia/impaired cognition group were generally older, less educated, more likely to belong to racial/ethnic minority communities, more often single, and more likely to live in rural areas. Older adults with dementia also utilized a higher proportion of hospital, hospice, and nursing home care, had fewer low and medium-level doctor visits, and exhibited increased frequencies of both high and no doctor visits compared to those with normal cognition.

Overall, our findings revealed significant trends in healthcare utilization. Older age was associated with increased utilization across various healthcare services and cognition levels. Hispanic ethnicity predicted fewer hospital stays in both groups and fewer doctor visits in the normal cognition group. Gender influenced hospital stays in dementia/impaired cognition group, nursing home stays in the normal cognition group, and doctor visits in both groups. Marital status was a predictor of nursing home stays and doctor visits in both groups. Race influenced nursing home stays in the dementia/impaired cognition group and doctor visits in the normal cognition group. Education predicted doctor visits in the dementia/impaired cognition group. No ACP measures mediated the associations between the study variables and healthcare utilization. Given the generally low rate of ACP among older adults in the U.S., further focused studies are needed to understand these results. More specifically, studies are needed to explore the barriers to ACP adoption and the potential impacts of increasing ACP engagement on healthcare outcomes across subgroups.

Our analysis revealed several key trends in healthcare utilization. The Hispanic population tends to use less healthcare, likely due to lower access to healthcare services and other discriminatory factors (Fernandez et al., 2023; Mcmaughan et al., 2020; Rahemi & Williams, 2020). Older age was associated with higher healthcare use, which is understandable given the increase in age-related comorbidities. Married individuals used fewer long-term care services, such as nursing home stays, but had more outpatient care, such as doctor visits. The reduced use of long-term care services among married individuals may be attributed to the familial and social support they receive, which influences their preferences and allows them to remain at home for a longer period (Mah et al., 2021; Rahemi & Williams, 2016).

Additionally, higher education significantly increased hospice care utilization, independent of ACP measures. This can underscore the influential role of education in healthcare decisions. We previously assessed the impact of social determinants of health on healthcare utilization in a 2014 cohort of HRS respondents; however, we did not account for ACP. We found that individuals in the impaired cognition/dementia group used more hospital and nursing home care, with either a high number of doctor visits or none at all and were less likely to have low to medium levels of visits compared to the normal cognition group (Rahemi et al., 2023b). As demonstrated in the current study with the 2018 dataset, where we accounted for ACP, we observed that compared to the normal cognition group, participants in the dementia/impaired cognition group used more hospital, hospice, and nursing home care, had fewer low to medium doctor visits, and showed higher frequencies of both high and no doctor visits. Fernandez and colleagues emphasized that diagnosis, disease awareness, and healthcare utilization are interrelated (Fernandez et al., 2023). They introduced the concept of the Hispanic health paradox: They reported that despite having lower income, education, and health insurance coverage compared to non-Hispanic Whites, Hispanics in the U.S. often experience equal or better health outcomes, such as higher life expectancy. This contradiction between socioeconomic disadvantage and better health outcomes is known as the Hispanic health paradox, which needs further consideration in related studies.

Our results align with prior research, suggesting that disparities in healthcare persist, particularly concerning socioeconomic factors like age, race/ethnicity, and education (Brown et al., 2016; Mcmaughan et al., 2020; Rahemi et al., 2023a, 2023b; Rahemi and Jarrín, 2023).

From Andersen Behavioral Model, we examined predictive variables mapping to the predisposing factors from both the contextual (rurality, neighborhood cohesion, neighborhood social score, discrimination score, living alone) and individual (race, ethnicity, education, gender, marital status, chronic conditions, loneliness, and depression) domains of the model. All of our outcomes for analysis were mapped to the health behaviors domain in the use of personal health services and process of medical care factors. As the Andersen Model suggests, these domains are related to each other both directly and indirectly impacting the process of and use of healthcare services (Andersen, 1995). Within the context of dementia, these factors impact racial/ethnic minority populations by making them less likely to receive timely and accurate diagnoses, be prescribed anti-dementia medications, or use hospice care. They are also more likely to face a higher risk of hospitalization and receive more aggressive life-sustaining treatments, even at the end of life (Hinton et al., 2024). In a study using Medicare claims, it was shown that Black beneficiaries faced higher risks of all-cause hospitalization, longer stays in skilled nursing facilities and shorter stays in hospice care compared to White beneficiaries. They were also less likely to receive physical/occupational therapy, dementia medications, and Parkinson’s disease medications (Lusk et al., 2023). These results highlight the disparity, especially considering that racial and ethnic minorities require more healthcare due to the disproportionate prevalence of dementia and comorbidities within these populations (Alzheimer’s Association, 2024; Charron-Chénier & Mueller, 2018). However, based on the Hispanic health paradox, there is a need to examine within-group differences among populations, as health outcomes and healthcare utilization vary by factors such as birthplace, immigration status, socioeconomic characteristics, and geographical context. Disaggregating the subgroups is essential to identify the underlying mechanisms driving disparities, particularly among older adults and those with chronic conditions. Understanding these differences is critical for developing targeted interventions to reduce preventable costs and improve care (Fernandez et al., 2023).

As societies become increasingly multicultural and witness an unprecedented aging of their populations, the implications of our findings are significant. With the rising number of individuals affected by ADRD and the aging baby boomer population, continuous research on healthcare utilization in this group is critical. This research is vital for identifying trends, disparities, and developing tailored policies to meet the unique needs of diverse and minority communities. Our results underscore the importance of developing culturally tailored care management programs and policies that improve resource allocation in dementia care and advance care planning. By focusing on these areas and understanding the values and dignity of diverse older adult populations, we can work towards reducing health disparities and improving the overall quality of care for older adults with dementia.

### Strengths and Limitations

A key strength of this study is its use of a large, nationally representative sample of older adults from across the U.S. However, several limitations must be considered when interpreting the findings. First, secondary data analysis is constrained by the available data and measurement tools in the dataset, limiting researchers’ control. Second, the HRS study design results include an overrepresentation of African Americans, Hispanics, and Florida residents. To ensure accurate and unbiased estimates, we used survey weights for descriptive statistics but did not apply them in the modeling analyses, following established academic practices. Third, our study lacks within-group differences, which necessitates further research to understand healthcare use within different racial groups. Fourth, although our analysis included a range of sociodemographic and health factors, other variables that may affect healthcare use, such as income and healthcare insurance, were not incorporated.

## Conclusion

Our study underscores the need for more research and policy development to address the healthcare disparities faced by an increasing number of diverse older adults, particularly those with dementia. As the aging population grows, it is crucial to understand the healthcare utilization patterns and disparities among diverse and minority communities. Our findings emphasize the importance of creating culturally tailored care management programs and policies that cater to the specific needs of racially and culturally diverse groups. By identifying factors that influence healthcare utilization, we can develop targeted interventions to reduce disparities and improve the quality of care for older adults with dementia. Respecting these populations’ values, desires, and dignity is vital for designing effective healthcare strategies and ensuring equitable access to healthcare services.

## Data Availability

The data generated for this study are included in the article. The HRS 2018 dataset used in this study can be accessed via HRS Public Survey Data. Multiple products of HRS are available. Public Survey Data are available for all registered users and was used for this study. Access to restricted and sensitive data needs special agreements.
